# An eye tracking study of the application of gestalt theory in photography

**DOI:** 10.16910/jemr.16.1.5

**Published:** 2023-03-31

**Authors:** Hsien-Chih Chuang, Han-Yi Tseng, Da-Lun Tang

**Affiliations:** Chinese Culture University, Taiwan; Huafan University, Taiwan; Tamkang University, Taiwan

**Keywords:** Photography, gestalt, eye tracking, closure, visual perception

## Abstract

Photography is an art form where integration of the human visual perception and psychological experiences result in aesthetic pleasure. This research utilizes eye tracking to explore the impact of the properties of Gestalt in photography on people's visual cognitive process in order to understand the psychological processes and patterns of photography appreciation. This study found that images with Gestalt qualities can significantly affect fixation, sightline distribution, and subjective evaluation of aesthetics and complexity. Closure composition images seem to make cognition simpler, resulting in the least number of fixation and saccades, longer fixation duration, and more concentrated sightline indicating stronger feeling of beauty, while images which portray similarity results in the greatest fixation and saccades, longest saccade duration, and greater scattering of sightline, indicating feelings of complexity and unsightliness. The results of this research are closely related to the theories of art and design, and have reference value for photography theory and application.

## Introduction

Photography is a visual art that emphasizes visual perception. It integrates human vision and psychological experiences to ultimately bring aesthetic pleasure. Photography is coming closer to the lives of people as an art form that is gradually becoming an essential means of recording daily life. In this age of heavy reliance on images as the primary medium, ‘Ways of Seeing’ by Berger (5) is particularly profound today. He mentioned that seeing comes before words, implying that a visually-based society is very different from a language-based one. After all, humans know how to see before learning to speak. Everyone in modern times is also a consumer of photography. Photographer Moholy-Nagy (41) once said that the illiterate in the future is a person who does not comprehend photography. However, we have very limited knowledge of how to watch and interpret a piece of photography when it is presented to us, and this, unfortunately, may move the critique and implementation of art education towards subjective creation, making this a fascinating issue. Composition is the science of placing and arranging the elements of a picture. Yarbus (60) pointed out that artists make it easier for viewers to perceive pictures through skillful application of composition. Studies by Beelders and Bergh (4) and Sancarlo et al. (50) all found that composition does affect visual perception, can attract the viewer's attention, and effectively guide the viewer's eyes along a predetermined path, making composition a significant component of photography.

Eye movement analysis technology is an important tool in psychological research. At present, eye tracking is matured technology that is widely used in the research of human cognition. Its application include usability engineering, art science, film experience, etc. (12; 43; 52). Eye movement analysis is a prime research method in psychological research and widely used in the field of applied psychology. Ma and Chuang’s (36) research has revealed that enclosed-form Chinese character structures, according to Gestalt theory, can indeed generate significantly different gaze patterns that are more concentrated, as compared to open-form character structures which generated more scattered patterns. This study intended to use the quantitative research methods of cognitive psychology with photographic work depicting clear visual structures in order to understand and verify the consistency of the finding. To this end, this study conducted an eye tracking-based psychological experiment to investigate the process of viewing photographic works depicting Gestalt principles by revealing differences with fixation frequency, gaze distribution, and subjective critique In order to understand the psychological process and patterns when viewing a piece of photographic work. The author is of the opinion, as an image creator, that Gestalt theory, other than appearing in common image compositional practices, is rarely mentioned in psychology studies on photography, and even fewer researchers have tried to integrate the two fields of art and cognition. It is therefore the intention of this research to explore, from the perspective of the important theory of Gestalt psychology, the visual presentation of Gestalt in photographical image composition, in hopes of discovering artistic inspirations of greater creative value, expand modern aesthetic theory, and invigorate artistic application.

## Literature Review

### Gestalt theory

The roots of Gestalt psychology go back to Gestalt Theory which was mainly established with the research results of three psychologists, Max Wertheimer, Wolfgang Köhler and Kurt Koffka. Gestalt psychology has been one of the main schools of modern western psychology. It contains the underlying laws of visual perception based on structural properties of the visual stimuli. Gestalt psychology investigates the process with which the human eyes identify and integrate the whole and the parts of things, whereby the whole is not equal to the sum of the parts, and consciousness is not simply a collection of single sensory elements, but rather determined by the characteristics of the whole (31; 57, 58). The principles of Gestalt play also an important role in visual aesthetics (2, 3; 20). Aesthetic theory has long proposed that balance, contrast, and clarity are objective determinants of beauty (21, 22; 27; 28; 38; 53; 59). The school of Gestalt Psychology advocates that the goodness of perceptual stimulation depends on the relationship between the stimulus organization and mental function (31). The proximity, similarity, and completeness perceived by the sensory system are underlying principles of graphic design and artistic painting, and their application will naturally produce pleasant feelings in the beholder.

There have been examples of theory integrated into a photography curriculum in academia, such as Lu's (35) empirical research on photography teaching, showing that Gestalt theory has good efficacy for the education of photography. Zakia's (64) theoretical teaching focuses on the application of the principles of Gestalt psychology and semiotic theory to enhance the photographer's ability in visual expression. In fact, a photograph that is attractive must have an explanation for these qualities. Gestalt psychology may help inexperienced photographers to overcome the problem of poor image composition (11).

Han and Cheng (23) stated that past works of photography had followed no theoretical basis and was completely dependent on the subjective expression of the creator. Most academic materials in photographic education are rather descriptive, not based on empirical research, reporting mostly techniques and technological application in photography (35; 42; 47).

**Figure 1.**
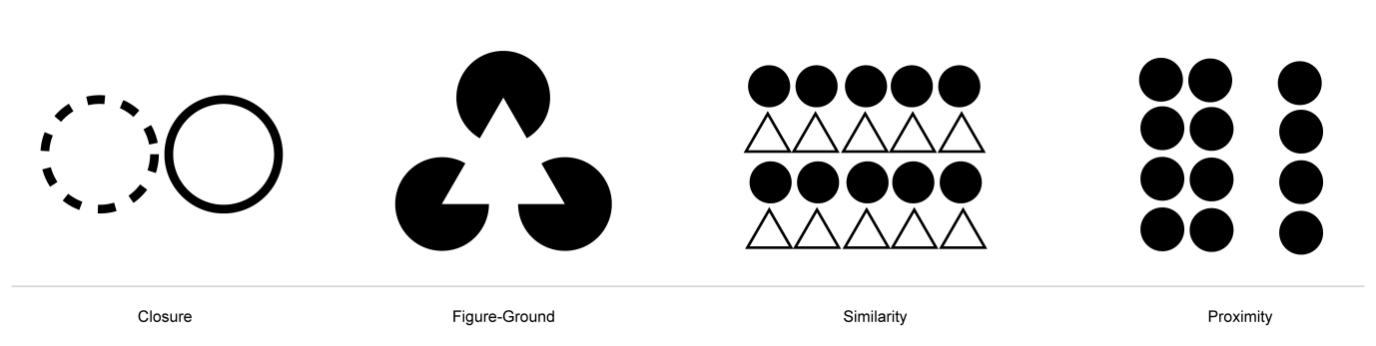
Illustrations of principles of gestalt (64)

In the following we will present the principles of Gestalt theory relevant to our research: (see Figure 1).

### Principle of closure

This principle refers to the tendency for image completeness. Photographic composition without closure can indirectly exploit this principle by creating incomplete and gapped images which might generate feelings of simplicity, relaxation, liveliness, and freedom, allowing more space for imagination, and avoid aesthetic fatigue from complete enclosure.

### Principle of similarity

We have a tendency for forms that display consistency and coherence, and this tendency makes us see elements with similarities in shape, size, color, attribute, motions, vectors as related elements in a unified whole. Viewing them as clean, symmetrical, orderly, and complete images can lead to the feeling of relaxation and pleasantness. We expect that when applying this principle in photography, the feeling of visual comfort and unity can be elicited, resulting in the impression of a more vivid, rhythmic, and charming image.

### Principle of proximity

Kepes (29) expressed that the less distance between certain visual elements, the more likely they are to relate to each other and generate overall unity. The more closely the photographer places the visual elements in the image, the stronger will be the association of the elements leading to a feeling of unity. This principle can be applied to concise and simple backgrounds in order to highlight the theme and increase overall aesthetics of the image.

### Principle of figure-ground

The separation of some part of the image into figure and the rest into background is a basic principle in human perception. Obvious boundaries and distinct features can often be formed between the two, a prime example being the Rubin vase (54). Increasing figure-ground contrast makes recognition faster and easier (9; 46).

## The Relationship Between Photography and Principles of Gestalt

Gestalt theory reveals the visual logic of human eyes observing images - the tendency towards gestalt, to decipher complexities into points, lines, and surfaces, and, in photographic image composition, to use rules of the relationship between the figure and the background to process the relationship between the main object and its surroundings, to use the property of closure to generate order, to use similar elements to adjust the pace of the image and properties of continuity and proximity to regulate the lines and spacing, and so on. Gestalt theory emphasizes the holistic view of the mind. Gestalt psychologists summarized and categorized a number of well-known principles, including proximity, similarity, continuity, closure, etc. Chiang (10) noticed that the photos of beginner photographers generally have the common problems of sloppy composition, vague subject and poor organization. He believes that the principles of Gestalt is an effective prescription for curing these symptoms, but also reminds that Gestalt theory is not a formula to be followed during creation. Zakia (62, 63, 64) used Gestalt theory as the basis of education in photography, and stated that the concepts of figure and ground, simplification, and grouping principles in Gestalt theory help to understand and describe more clearly the composition of images or the visual syntax in a photograph. The Gestalt principles themselves are not for the purpose of creation, and are instead best regarded as means to an end that guides photographers to create more convincing images that effectively convey visual information.

**Figure 2.**
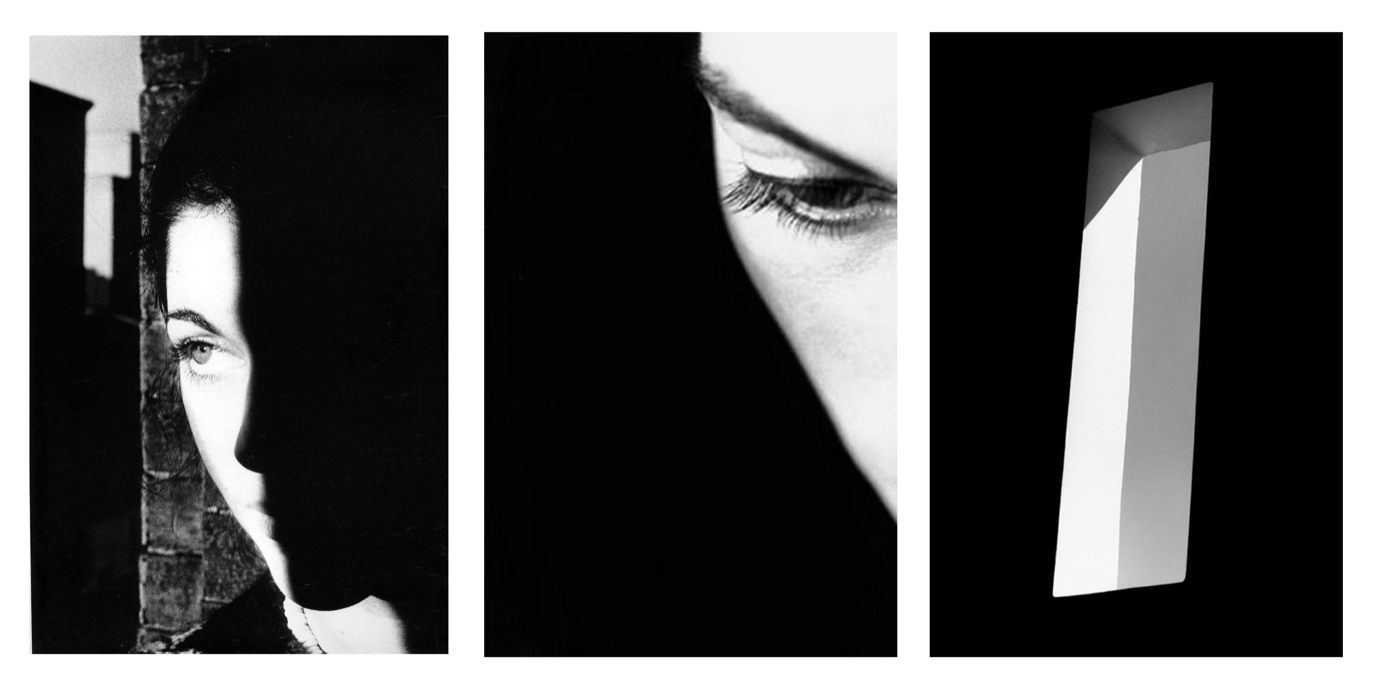
Examples of gestalt photographic works by Ralph Gibson

It can be discovered from works by famous photographers that visual expressions which can effectively grab people’s attention don’t necessarily need to be complex, as overly complex images can increase the viewer’s cognitive burden (30). Indeed, many timeless photographic works are visually quite straightforward. The famous contemporary photographer Ralph Gibson builds his particular way of photography around exploring imagery that convey single objects or themes with the use of overexposure technique to create a highly granular and highly contrasted visual style. His works are full of rich visual elements, and the simple imagery contains boundless emotions and sensations. His photographic works are often created using methods consistent with Gestalt psychology, allowing viewers to automatically form gestalt cognition when viewing these works (Figure 2).

### Eye movement

Eye movement can be used to understand visual perception which contains complex information; it began by studying static pictures in the early stages to later more complex and varying dynamic videos (15; 52). By observing the viewer’s eye reactions in the form of their gaze trajectories, we can directly infer the high-level cognitive processing of the brain and visual image perception (16; 44; 48). From static art viewing research in the past, Berlyne (6) put forward the concept of exploratory behavior. He stated that when viewing a painting for the first time, one usually goes through the following transition of explorative states: First the eye performs a series of large movements that browse, then the movements scale down and becomes slower in a particular way. The study of eye movement in art uses mainly fixation position and duration of the fixation as indicators. Earlier investigations of Buswell (7) and subsequent experiments by other researchers (e.g. 13; 55) emphasized that most of the gaze points were concentrated on the areas of interest. Yarbus (60) argued that the act of perceiving a painting is composed of a series of observation cycles, and each cycle has many similarities. In the process of appreciating a painting, the observer’s eyes often returns to an area of importance, and so, by analyzing the distribution of the gaze points, or fixation points, in each area on the image it is possible to identify the area of interest. Henderson and Hollingworth (25) argued that the two most important issues in observing eye movements are what area they are fixated on and the duration of the fixation there. Although internal interest and external eye movements are not completely matched, most researchers still agree that there is a strong correlation between the fixation position of the eyes and the area where attention is paid (1; 14; 18, 19; 25; 26; 36; 40).

### Fixation duration and number of fixations

There are two states of eye movement: periods of relative stability, called fixations, and those of rapid jerky movements, called saccades. However, it should be kept in mind that even during fixations the eyes undergo small saccades, called microsaccades which are necessary for perceptual processing (39) and are correlated with various cognitive processes (e.g. 32; 33; 51). The fixation happens when the brain is processing information (17). The number of fixation refers to the number of times this state occurs, and fixation duration refers to the length of time that the eye is temporarily still while gazing in a certain area, with the unit of calculation being milliseconds (ms). The duration of fixation may reflect the amount of cognitive processing during the fixation, but also personal preference or the complexity of external stimuli. As the information present increases in quantity and complexity, the fixation time is also increased (24; 37; 49). In particular, viewers will spend longer fixation time on emotionally stirring pictures than those that are more neutral (8).

### Number of saccades and saccade duration

As outlined above, the alternate state to the fixation is the saccade, and the visual processing is diminished during a saccade (39). And so, within a fixed amount of time, the number of saccades is proportional to the number of fixations, and the saccade duration is inversely proportional to the fixation duration. This can be used to reconfirm the stability of the fixation data obtained by the eye tracker.

### Spatial Dispersion Index (SDI) of fixation

Eye tracking research has one more indicator: the amplitude of saccade which can indicate attention span. However, because the rate of saccades is extremely fast, low-speed eye trackers cannot accurately ascertain this value. Instead, some researchers switch to using fixation dispersion to convey the average spread of attention across subjects. This is an index used by the Geographic Information System to describe the degree of fixation point dispersal (56). This type of analysis describes to what degree the fixation is dispersed or concentrated when viewing an image. Generally speaking, the more concentrated the fixation points, the lower the degree of dispersion and the smaller the SDI. Conversely, the more scattered the fixation points, the higher the degree of dispersion and the greater the SDI (36). The formula is as follows:

**Figure:**
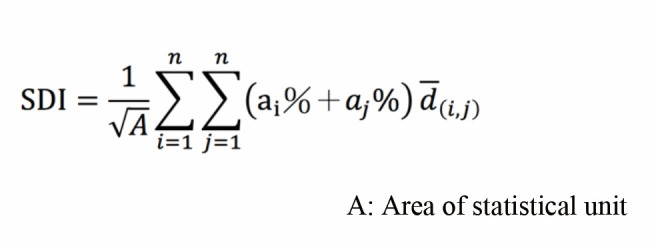


### Hypothesis

Sancarlo et al. (50) found that composition of pictorial elements affects visual perception. In the past, photographers mostly produced work from their own subjective perspective while the influence of the gestalt structure on the way people view them remains neglected. If the photographer can simultaneously grasp how to change image composition characteristics and understand how images are perceived and the visual cognitive process involved in each work, it will not only effectively help creators grasp the ability to manipulate image expression, but also allow viewers to quickly comprehend the photo. Therefore, we investigate different gestalt compositional rules and observe the psychological experience and sightline characteristics of the average person for these compositions. Based on the aforementioned literature, the following hypotheses can be established:

H1: The closure property in the closure and figure ground contrast composition will make the sightline more concentrated and reduce the number of times fixation point changes.

H2: Closure and figure ground composition images conform to closure properties and will be the simplest and most aesthetically pleasing in subjective evaluation.

Supposing that a photographic work is being viewed, if the image composition has a significant impact on the area of interest and viewing behaviour, then it can be expected that when viewing other images of the same composition, the eyeballs will have the same fixation state, saccade changes, and dispersion changes. This study will analyze the changes of the above-mentioned eye movement characteristics through complex multivariate statistical methods, in hopes to establish objective change indicators, further the evaluation of the true utility of photography creation, and become beneficial to the teaching and learning of photography and visual arts education.

## Methodology

### Experimental design

**Figure 3.**
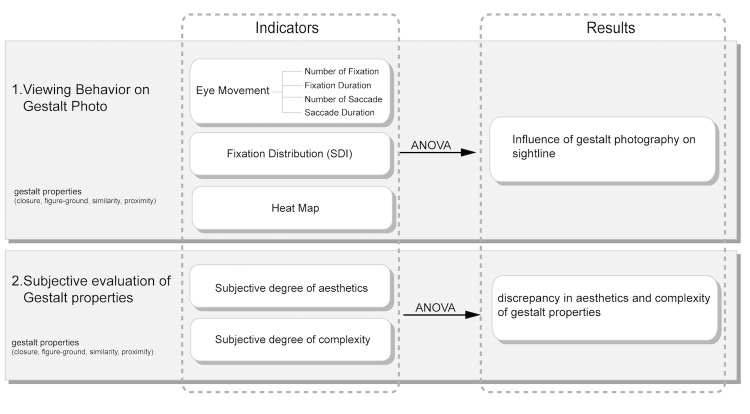
Research framework

The main purpose of this research is to employ eye tracking to explore Gestalt principles in photography in order to observe the influence of photographic composition inspired by Gestalt theory on eye movement characteristics and gaze distribution. Figure 3. is the design of the experiment.

### Participants

A sample of 8 males and 25 females aged between 18 and 25 were recruited resulting in a total of 33 participants. All participants were college students, native speakers of Mandarin, had normal or corrected to normal vision, participating in the entire experiment. All stimuli were presented in random order in a within-subject design to every participant.

### Experimental stimuli

**Figure 4.**
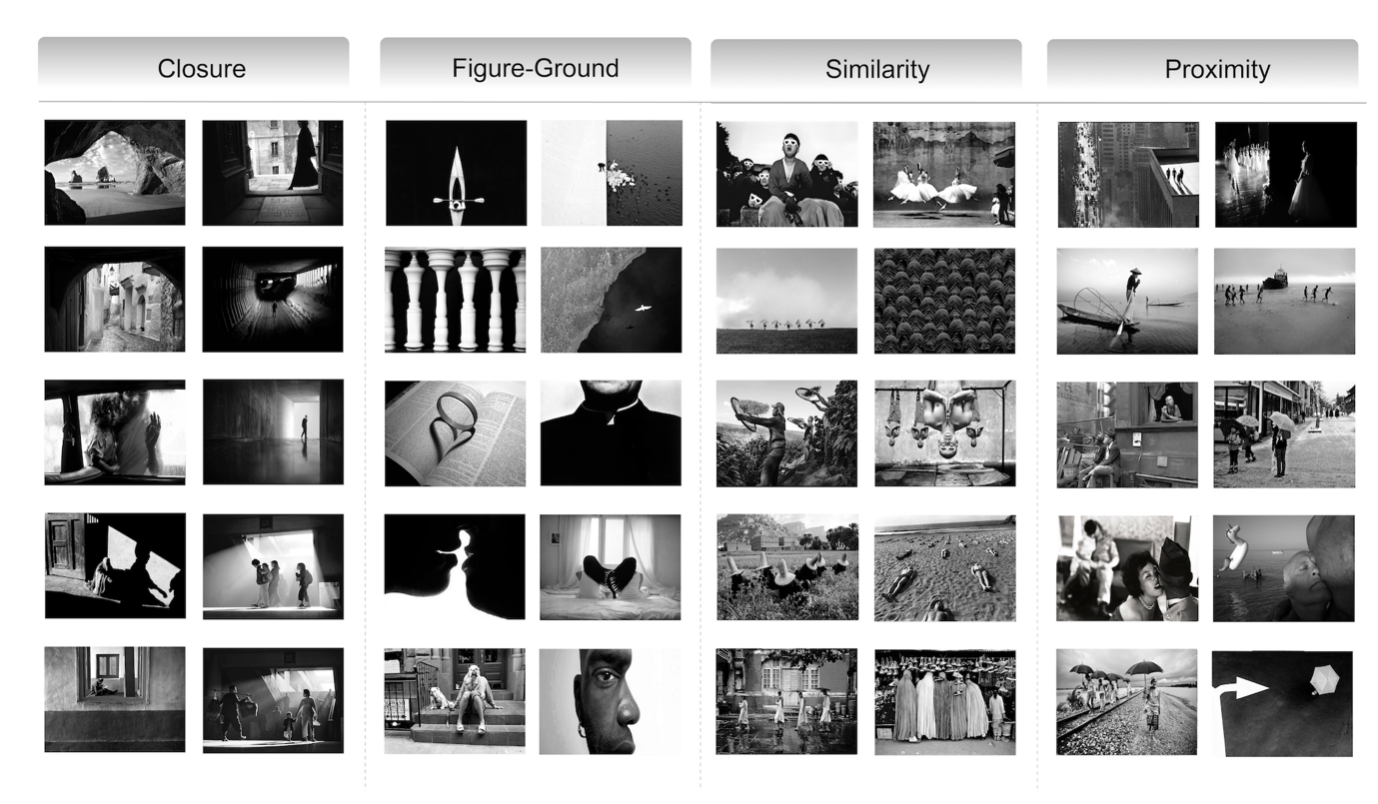
Stimulus material

A focus group interview method was used to validate the criteria of the experimental photos. Four senior photography experts and scholars, each with more than 15 years of practical and teaching experience, were invited to conduct interviews. Before the interview, the photo works of internationally renowned photographers were preselected, and the four experts evaluated and selected in group discussions the photos that conformed to the gestalt criteria. The experimental pictures should follow the rules summarized by gestalt psychologist Zakia (64). The four gestalt properties investigated in this study were closure, figure-ground, similarity, and proximity, as shown in Figure 1, with 10 representative works for each category selected by the experts. A total of 40 photos with 1920*1440 pixels resolution were used (Figure 4).

Experimental manipulation:

Independent variables: Gestalt properties (closure, figure-ground, similarity, proximity)

Dependent variables: Eye movement characteristics (Total number of fixations, Total fixation duration, Number of Saccades, Saccade duration, Spatial Dispersion Index (SDI) of fixation, Aesthetics, Complexity)

### Experimental procedure

**Figure 5.**
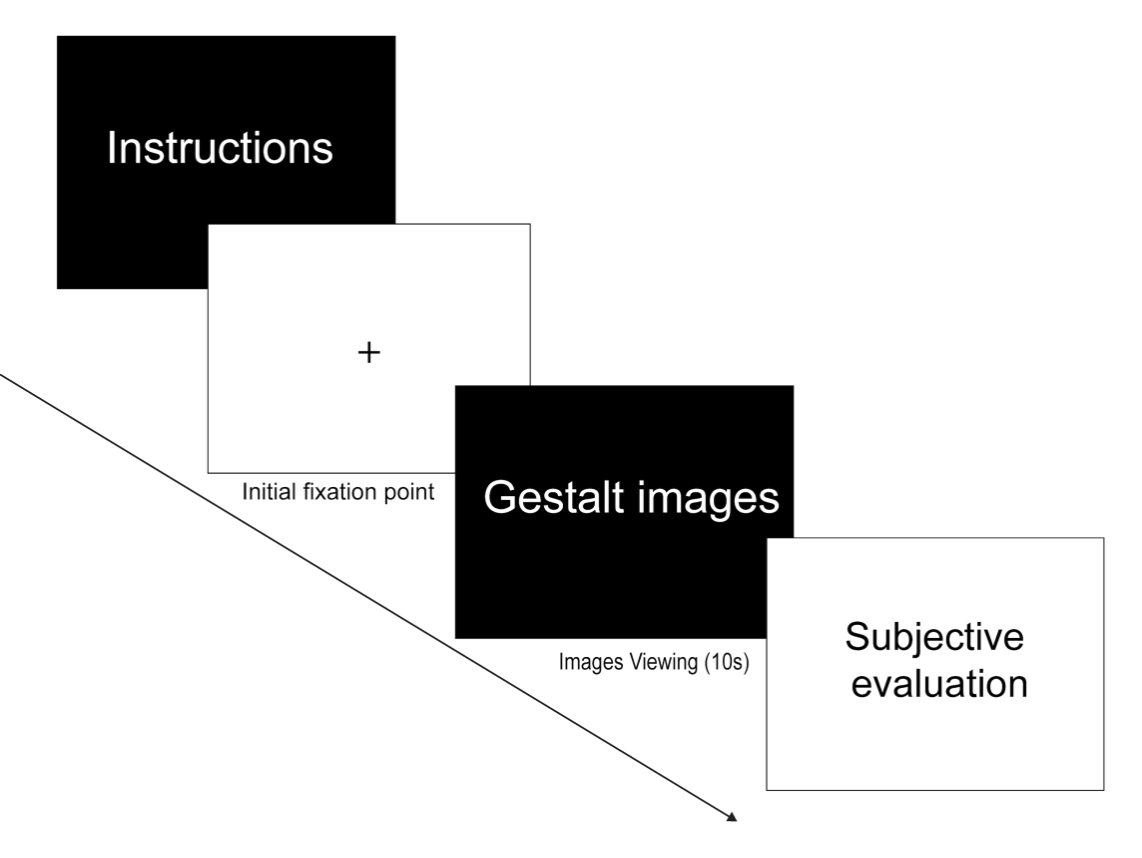
Flow chart of experimental procedure

Before the experiment, each participant was placed 60 cm in front of a 21-inch CRT screen, the center of the screen in line with the participant. The eye tracker (Tobii Pro Nano) was set to record the gaze trajectory at a sampling frequency of 60Hz, followed after a 9-point calibration. At the beginning of the experiment the instructions were read and a series of practice trials were conducted to familiarize the participants with the experiment. The participant was asked to move the mouse cursor to the center of a cross symbol on the screen to trigger the display of the photographs. The subsequent gaze trajectory during the viewing process was recorded in full. Each photo image was displayed for 10 seconds in random order. This sequence was repeated a total of 40 times to complete the full set of photo images. At the end of the presentation of each stimulus the participants were asked to score the subjective psychological complexity and aesthetics of the 40 photos on a Likert scale ranging from 1 to 5, five being very pleasing for aesthetics, and 1 to 5 for complexity from being very simple to very complex. The full experimental session for each participant lasted approximately 10 minutes. Figure 5 outlines the experimental procedure.

## Results and Discussion

### The effect of gestalt properties

**Figure 6.**
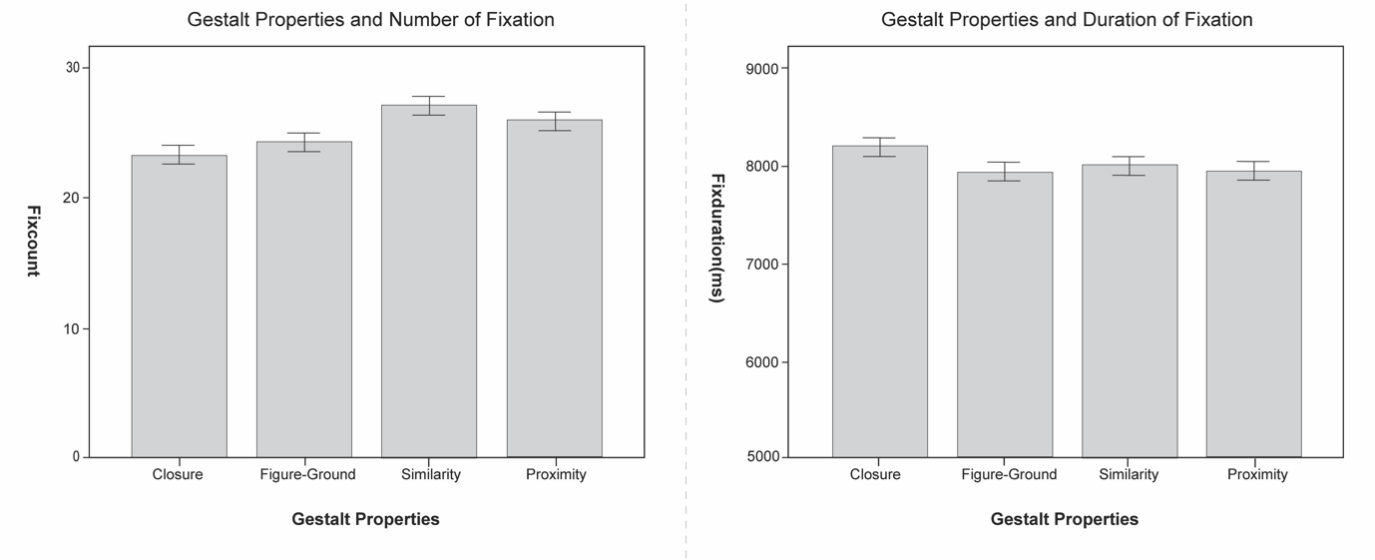
The Number of fixation and fixation durations of different gestalt properties

In the ANOVA of the dependent variables total number of fixations and total fixation duration, it was found that gestalt properties have significant main effects on the number of fixations (F(3, 1188) = 14.952, p <0.01) and fixation duration (F(3, 1188) = 7.565, p<0.01), and do affect the overall gaze distribution. In particular, images with property of closure have the smallest number of fixations and the longest viewing time, and are therefore more complicated and take the most time to view, particularly, photographs with similarity property obviously have the highest number of fixations, which means that this type of photograph is more complex and requires more effort to comprehend, as shown in Figure 6.

**Figure 7.**
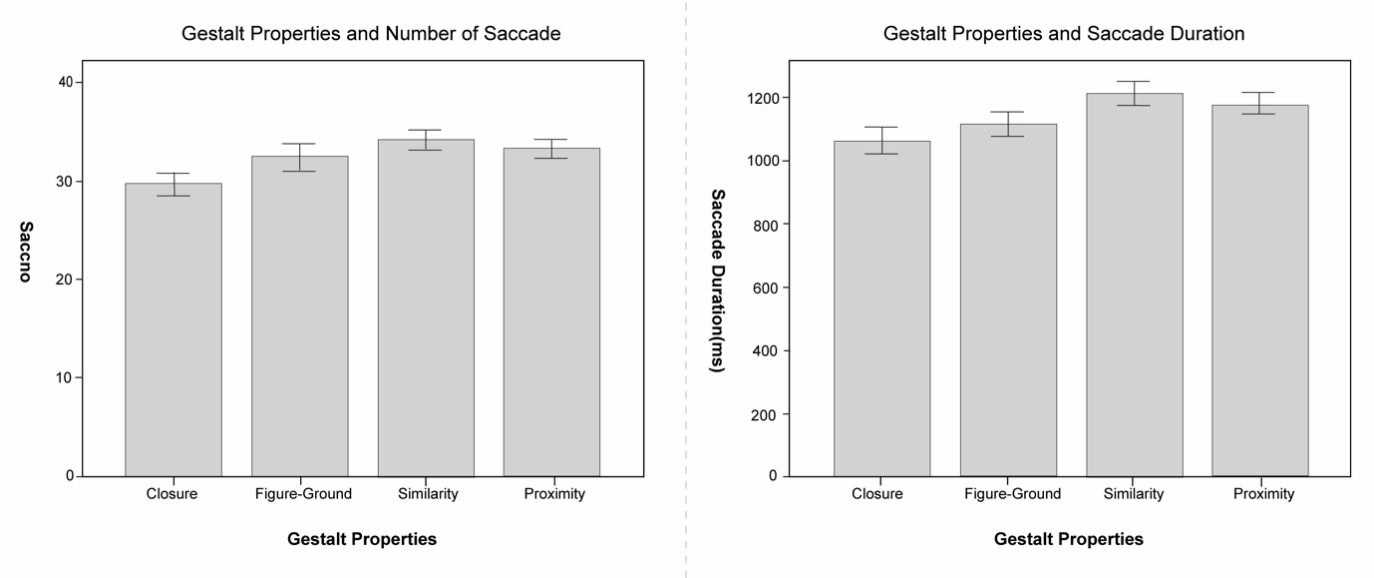
The Number of saccades and saccade duration of different gestalt properties

In the ANOVA of the total number of saccades and the total saccade duration dependent variable, the results showed that the gestalt properties have significant primary effects on the number of saccades (F(3, 1188) = 6.771, p <0.01) dependent variable and saccade duration (F(3, 1188) = 7.002, p <0.01). Images with similarity property have obviously higher number and longest duration of saccades, implying that content with a high degree of similarity is more complex and less easy to view. In contrast, closure property images facilitate viewing, as shown in Figure 7.

**Figure 8.**
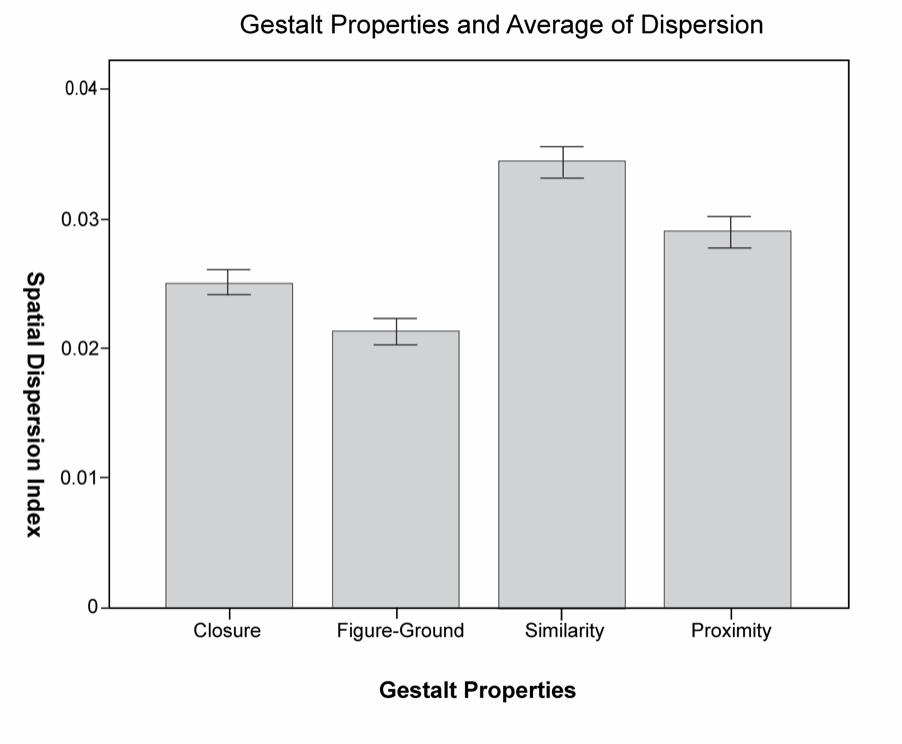
Dispersion of different gestalt properties

The SDI of fixation reflects the breadth of the fixation distribution on the photo images from the ANOVA analysis of the variance of the gestalt form and the fixation dispersion. Data shows that the gestalt properties have significant primary effect on (F(3, 1440) ) = 186.955, p <0.01) on the dispersion as a dependent variable. Apparently, the degree of fixation dispersion of different gestalt properties is different, and the degree of fixation dispersion of viewing photos is different, as in, the sightline of viewing closure and figure-ground types of photos are more concentrated. Conversely, photos with similarity property have a higher degree of dispersion SDI, and the fixation distribution is relatively more scattered, as shown in Figure 8.

**Figure 9.**
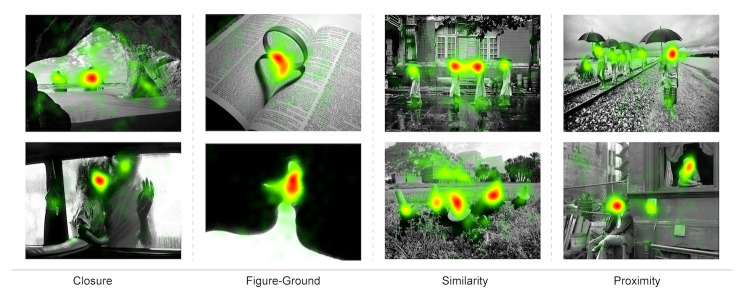
Heat map representation of gaze distribution

It is difficult to quantify how the viewer will look at a particular image, or which type of photographic work is better at grabbing attention. However, by studying the information from the vision in the eye movement data, researchers can begin investigation into the stimulus material (45). Through looking at appropriate changes in the fixation characteristic value, the dynamics of the viewing process can be outlined to construct a universal and quantifiable viewing mode. Smith and Henderson (52) demonstrated that heat map analysis is able to visualize the viewers’ attention distribution. For this purpose, this study adopted a precise dispersion indicating heat map consisting of multiple subject data sets to qualitatively investigate gestalt-based image structure and visual communication. Heat analysis of each gestalt property as distribution can be visually presented in this manner, which reveals areas of interest and their dispersion level. The heat map visualization of multiple subject data sets of areas of interest are shown in Figure 9.

### The effect of gestalt properties in images on subjective evaluation

**Figure 10.**
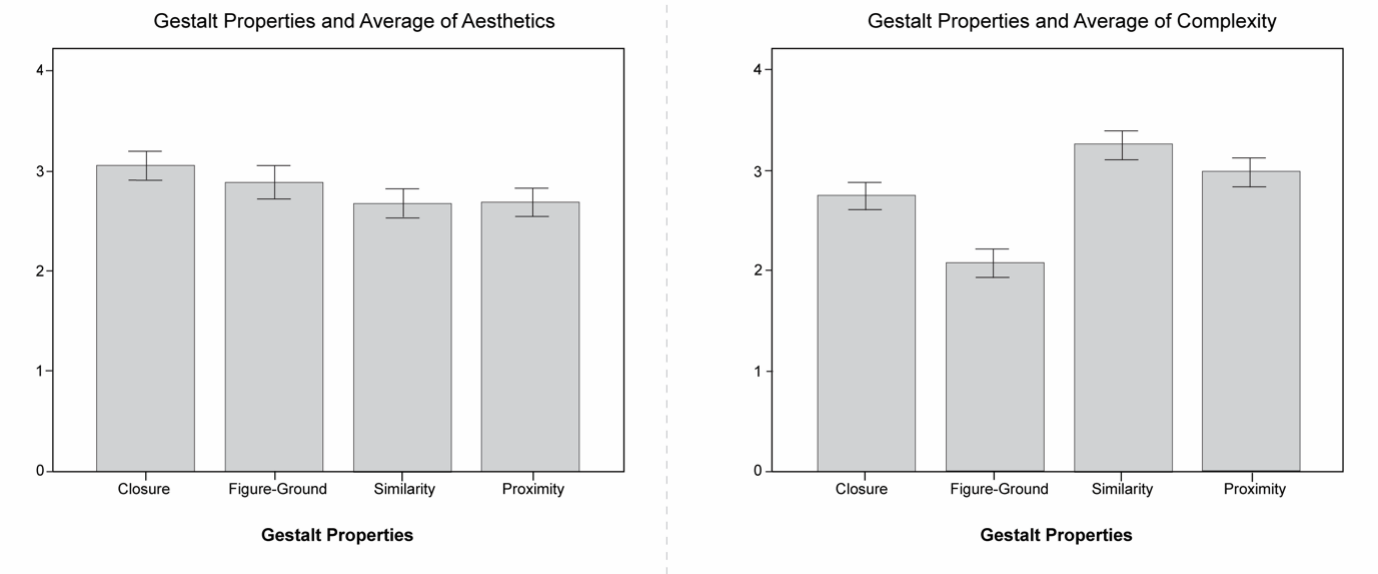
The aesthetic and complexity score of different gestalt properties. Photos of similarity are most complex and have the lowest aesthetic score

Subjects were asked in the subjective experience portion of the experiment to give scores for complexity and aesthetics. Analysis revealed that gestalt properties have significant primary effect on aesthetics (F(3, 1188) =3.646, p <0.05), indicating that different gestalt properties influence the ratings of aesthetics, as shown in Figure 10. The analysis further shows that the type of gestalt property also has a significant main effect on feeling of complexity (F(3, 1188) =30.452, p <0.01), which means that different gestalt properties induce different feelings of complexity for the viewers, particularly with similarity and proximity properties which scored clearly higher in complexity and lower overall in aesthetics. In addition, figure-ground and closure property photos scored higher in subjective aesthetics and lower in subjective complexity. This study also found a positive correlation between the subjective evaluations of complexity and aesthetics.

This study employed properties of gestalt structure, calculated the Spatial Dispersion Index (SDI) of fixation, and collected the viewer's subjective aesthetic evaluation of several pieces of photographic work. From the analysis results of objective eye movement characteristics and qualitative heat map, the comprehensive results of this study revealed that photos of different gestalt properties do affect the overall eye movement characteristics during viewing, particularly photos with closure property which had different results from other types (such as Table 1). This study indicates that, with images conforming to the principle of closure, similar to Chinese characters in a previous study (36; 61), when compared to images with multiple focal elements such as those with properties of similarity and proximity, number of fixations was significantly lower, and fixation duration was significantly longer, due to the simpler focal point composition, resulting in an easier level of cognition and being more able to concentrate the sightline for a stronger sense of aesthetic beauty. This result is similar to the research findings of Liu et al. (34) who found, when participants were looking at Japanese gardens, they showed more ocular activity and higher degrees of physiological activation when viewing attractive Information.

**Table 1:**
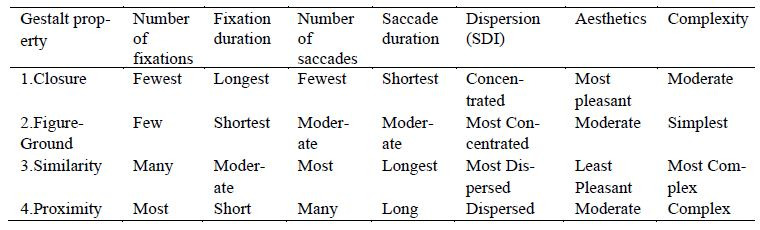
Comprehensive Analysis of Gestalt Photography and Eye Movement Characteristics

For image compositions with property of similarity, however, fixation and saccades were most frequent, saccadic duration was longest, and sightline was most dispersed, indicating that the viewer was constantly making comparisons, and thus required more time and mental effort to keep attention on the photograph for multiple repetitions. The two indicators of subjective complexity and aesthetics also reflect the viewer’s subjective psychological perception of the form, which indeed indicate that similarity-type composition were evaluated to be more complex and least aesthetic, and figure-ground-type compositions are instead less complex and more stable. The visual system tends to consolidate towards enclosure and wholeness making it easier for the sensory system to perceive in entirety. This makes figure-ground-type composition exhibit the least amount of dispersion, or the most concentrated sightline patterns, scoring the least in complexity compared to other image composition types. This phenomenon is closely related to art design theory.

## Conclusion

Photography is an art form that wields a visual language. Photographic image composition is an important component of the creative process. The process of capturing, selecting, reorganizing, and constructing the image that exists in the frame is the way of thinking in photography creation as well as the process of visual cognition. Any form of image composition is closely related to visual perception. Visual perception is like the invisible skeleton upholding the composition and layout of a photographic image, reinterpreting photography from a new perspective. In the past, our research found that certain structures of some Chinese characters possess the gestalt property of closure (36). This research further uses psychological quantitative research to explore the gestalt properties of photographic composition, which was verified to have a concurring conclusion using image-based photographic works. The study found that photographic works with different forms of gestalt significantly affect the eye movement information during viewing. The photographic works of figure-ground and enclosed composition conform to the principle of closure, and viewers find them the simplest to recognize while pleasing to look at. However, similarity compositions images require time and effort, and are the most complicated and unpleasant, to look at. The affirming empirical results of this eye-tracking research have potentially effective application in art design and photography creation.

Professional photographers understand the laws of vision and Gestalt principles, and can effectively control the composition form in photographic creation. They also know that principles of figure-ground in gestalt theory and simplification enable the guiding of the viewing process. Furthermore, management of the pace and the theme of a photographic work makes for more precision in what the final work can confidently express and create a good piece of photographic end product, all for the sake of giving the viewer a better experience. It is hoped that this study can contribute to the understanding of the theoretical framework of imagery, allow for the combination of cognitive psychology and aesthetics, enable the laying of a solid theoretical foundation for the development of photographic art, and provide material for substantial photographic practice teaching references and applications.

### Ethics and Conflict of Interest

The author(s) declare(s) that the contents of the article are in agreement with the ethics described in http://biblio.unibe.ch/portale/elibrary/BOP/jemr/ethics.html and that there is no conflict of interest regarding the publication of this paper.

### Acknowledgements

This work was supported by the Ministry of Science and Technology of Taiwan [MOST 108-2410-H-034 -042]. The Institutional Review Board of National Taiwan University approved this study (IRB#: 201905EM121).
